# Diabetes Detection and Management through Photoplethysmographic and Electrocardiographic Signals Analysis: A Systematic Review

**DOI:** 10.3390/s22134890

**Published:** 2022-06-29

**Authors:** Serena Zanelli, Mehdi Ammi, Magid Hallab, Mounim A. El Yacoubi

**Affiliations:** 1 University of Paris 8, LAGA, CNRS, Institut Galilée, 93200 Saint Denis, France; mehdi.ammi@univ-paris8.fr; 2 SAMOVAR Telecom SudParis, CNRS, Institut Polytechnique de Paris, 91764 Paris, France; mounim.el_yacoubi@telecom-sudparis.eu; 3 Clinique Bizet, 75116 Paris, France; magid@axelife.com

**Keywords:** PPG signal, diabetes, ECG signal, machine learning, deep learning, glucose estimation

## Abstract

(1) Background: Diabetes mellitus (DM) is a chronic, metabolic disease characterized by elevated levels of blood glucose. Recently, some studies approached the diabetes care domain through the analysis of the modifications of cardiovascular system parameters. In fact, cardiovascular diseases are the first leading cause of death in diabetic subjects. Thanks to their cost effectiveness and their ease of use, electrocardiographic (ECG) and photoplethysmographic (PPG) signals have recently been used in diabetes detection, blood glucose estimation and diabetes-related complication detection. This review’s aim is to provide a detailed overview of all the published methods, from the traditional (non machine learning) to the deep learning approaches, to detect and manage diabetes using PPG and ECG signals. This review will allow researchers to compare and understand the differences, in terms of results, amount of data and complexity that each type of approach provides and requires. (2) Method: We performed a systematic review based on articles that focus on the use of ECG and PPG signals in diabetes care. The search was focused on keywords related to the topic, such as “Diabetes”, “ECG”, “PPG”, “Machine Learning”, etc. This was performed using databases, such as PubMed, Google Scholar, Semantic Scholar and IEEE Xplore. This review’s aim is to provide a detailed overview of all the published methods, from the traditional (non machine learning) to the deep learning approaches, to detect and manage diabetes using PPG and ECG signals. This review will allow researchers to compare and understand the differences, in terms of results, amount of data and complexity that each type of approach provides and requires. (3) Results: A total of 78 studies were included. The majority of the selected studies focused on blood glucose estimation (41) and diabetes detection (31). Only 7 studies focused on diabetes complications detection. We present these studies by approach: traditional, machine learning and deep learning approaches. (4) Conclusions: ECG and PPG analysis in diabetes care showed to be very promising. Clinical validation and data processing standardization need to be improved in order to employ these techniques in a clinical environment.

## 1. Introduction

Diabetes mellitus is a clinical condition that causes a high amount of glucose in the blood with a simultaneous lack of insulin or insulin resistance. Insulin is a hormone produced in the pancreas, which regulates the amount of blood glucose produced by the carbohydrates in the food we intake. This condition, also known as hyperglycemia, can damage the heart, blood vessels, eyes, kidney and nerves [1]. Diabetes can be caused either by the incapability of the body to produce enough insulin or by the resistance of the body to the insulin. As stated by the World Health Organization, about 422 million people have diabetes and 1.5 million deaths are directly attributed to diabetes each year [2]. Diabetes can be classified into three types: type 1, type 2 and gestational diabetes [3]. Type 1 diabetes is a chronic condition in which the pancreas produces little or no insulin. Different factors, including genetics and some viruses, may contribute to type 1 diabetes. Although type 1 diabetes usually appears during childhood or adolescence, it can develop in adults too. Type 2 diabetes is a chronic condition in which the pancreas does not produce enough insulin, and cells respond poorly to insulin and use less sugar. Type 2 diabetes used to be known as adult-onset diabetes but, as well as type 1 diabetes, it can begin during childhood and adulthood. As a matter of fact, the increase in the number of children with obesity has led to more cases of type 2 diabetes in younger people. Gestational diabetes is high blood sugar that occurs during pregnancy and usually disappears automatically after giving birth. It happens when the body cannot produce enough insulin to meet the extra needs in pregnancy. The related risks can be reduced if the condition is detected early during the pregnancy and well managed.

Being a chronic disease, diabetes cannot be cured. To minimize side effects and the worsening of the disease, early prevention and treatments, such as drugs or changes in lifestyle, are essential [4]. The development of new low-cost, non-invasive and light technologies that are able to detect the onset of diabetes could make a difference in large-scale prevention. With the advent of machine learning and deep learning, the processing of large amounts of data becomes easier and more effective every day. In the last years, these methods have been successfully applied to big health care data [5] in order to accompany the healthcare personnel through the decision process. Chaki et al. [6] propose a systematic review of machine learning, deep learning and artificial intelligence based methods applied to the detection and self-management of diabetes. The datasets that were used to detect and manage diabetes are varied and diverse. Electronic health record (EHR), voice analysis, weekly steps count, anthropometric characteristics and drug treatments, electrocardiography and photopletysmopragraphy analysis are only a part of the used datasets. A survey on diabetes detection with similar outcomes is proposed by Anwar et al. [7]. Zhu et al. [8] propose a review that focuses on the deep learning approaches to detect diabetes, detect diabetes complications and estimate blood glucose. Once again, the datasets used in the cited papers are composed of different types of data. For diabetes detection, the used datasets are mainly composed by EHR. Food intake, PPG signals and insulin levels have been used to estimate blood glucose. Among the diabetic complications, they mainly analyzed the presence of diabetic retinopathy with image classification and the intensive unit mortality rate care with PPG signals.

As shown in the previous paragraph, there are several data that can be used to detect and care for diabetes. Among those, photoplethysmography (PPG) and electrocardiography (ECG) signals offer several advantages. They are non-invasive and they can be easily embedded in wearable devices [9,10]. This offers, for example, the possibility to have 24 h analysis and real-time remote surveillance. As a result, there is an increasing number of papers that have been recently published. They employ PPG and/or ECG alone or in combination with other data, such as age, gender and BMI (body mass index). However, a review paper that exhaustively covers this domain has not been published yet. Gusev et al. [11] propose a review on machine learning and deep learning applied to heart rate variability (HRV) to estimate blood glucose. Swapna et al. [12] propose a review on diabetes detection with ECG signals going from traditional methods to deep learning methods. Both reviews are focused on the HRV analysis without taking in consideration all the other cardiovascular modifications related to diabetes. In addition, both of the cited reviews are mainly focused on ECG. However, PPG is largely employed in diabetes care, thanks to its cost-effectiveness and its simplicity of usage with respect to ECG. It is often used in blood glucose estimation and diabetes detection, while the analysis of diabetes complications is still not fully explored. Both ECG and PPG signals have been largely analyzed with traditional, machine learning and deep learning methods in diabetes detection and management. To our knowledge, no existing publication provides a detailed and comprehensive review of ECG and PPG signals analysis in diabetes care (detection of diabetes, glucose estimation and diabetes complication). We do believe that an extensive review on PPG and ECG signals analysis in diabetes care could be very helpful since most of the analyses used in diabetes detection, such as HRV and HR analysis, can be computed from both PPG and ECG signals [13,14,15]. In this review, we aim to provide an exhaustive overview of all the published methods, from the traditional to the deep learning approaches, to detect and manage diabetes using PPG and ECG signals. Particular attention will be given to the advantages and disadvantages of each method, employed data and employed features. This review will allow researchers to compare and understand the differences, in terms of results, amount of data and complexity, that each type of approach provides and requires.

The rest of the paper is organized as follows. In Section 2, we present the extraction procedure we follow to obtain all the articles included in this review. In Section 3, we briefly present the diabetes pathology, its complications and the conventional diagnosis tests. Section 4 is dedicated to the introduction of ECG and PPG signals. Results and discussion issues from the article’s review are presented in Section 5. In this section, we present a review of the traditional, machine learning and deep learning methods applied to PPG and ECG analysis to detect and manage diabetes and its complications. Finally, in Section 6, we conclude and discuss future work.

## 2. Methods

A systematic review is defined as a review of the evidence on a clearly formulated question that uses systematic and explicit methods to identify, select and critically appraise relevant primary research, and to extract and analyze data from the studies that are included in the review [16]. In this section, we present the methodology utilized to conduct this systematic review, based on the guidelines described in the PRISMA method: preferred reporting items for systematic reviews and meta-analysis [17]. The PRISMA 2020 guidelines for systematic reviews [18,19] are followed in order to achieve a valid formulation in this study.

### 2.1. Eligibility Criteria

The eligibility criteria for the selection of articles are listed below:

- Only papers written in English;
- Only papers with diabetes detection, glucose estimation and diabetes complications detection as the main topic;
- Only papers dealing with the management of type 1, type 2, gestational diabetes or diabetes complications;
- Only papers that focus on diabetes care through ECG and/or PPG analysis;
- Only papers addressing the topic with traditional methods, machine learning or deep learning methods.

Electronic searches were performed by S.Z. No date range was used, ensuring that no restriction was placed on the date of publication. All publications identified were screened for inclusion. Papers were excluded if they (i) were a short conference/congress abstract, or (ii) were not available in full text.

### 2.2. Data Sources and Search Strategy

We performed articles extraction on PubMed, Google Scholar and IEEE Xplore. The search strategy combined the following terms: PPG signal, ECG signal, diabetes detection, blood glucose estimation, diabetes complications, machine learning and deep learning (PPG signal AND diabetes detection, PPG signal AND blood glucose estimation, PPG signal AND diabetes complication, PPG signal AND diabetes detection AND machine learning, PPG signal AND blood glucose estimation AND machine learning, PPG signal AND diabetes complication AND machine learning, PPG signal AND diabetes detection AND deep learning, PPG signal AND blood glucose estimation AND deep learning, PPG signal AND diabetes complication AND deep learning).

### 2.3. Study Selection

**Figure 1:**
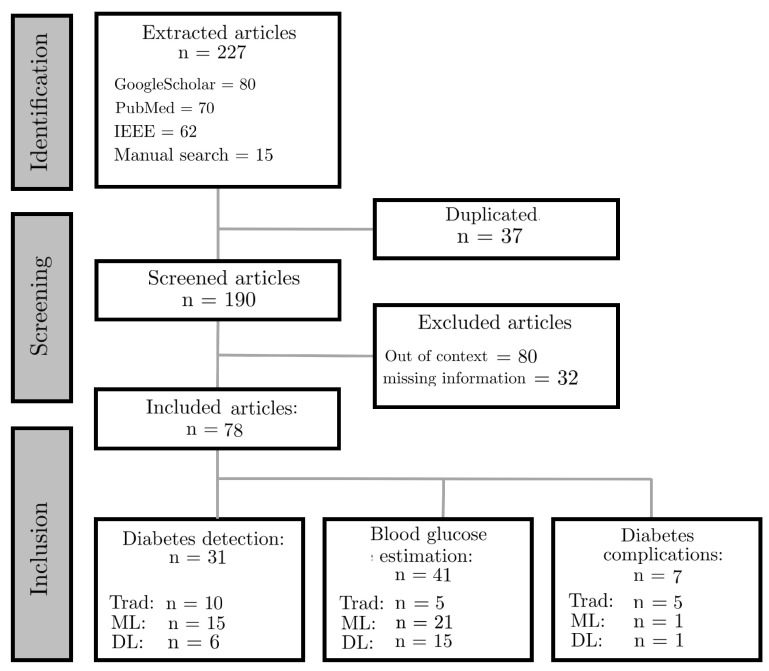
The PRISMA flow diagram. ML: machine learning approaches. Trad: traditional approaches. DL: deep learning approaches.

After querying the databases, we used the Mendeley reference management tool [20] to log references, eliminate multiple records, and create a unique database of references. To select articles from the initial database, we applied a three-step process: (1) evaluation of the title; (2) evaluation of the abstract and keywords; and (3) evaluation of the full text. The aim was to remove irrelevant searches during stages (1) and (2), and then review the remaining documents using the above eligibility criteria during stage (3). The entire flowchart for the selection process, including identification, screening and inclusion, is shown in Figure 1.

### 2.4. Data Extraction

For each article included, the following information was also included: (i) study objective, (ii) type of signal, (iii) length of signal, (iv) number of participants, (v) availability of the database, (vi) approach used, (vii) extracted features, and (viii) main outcomes. The results were discussed in order to show advantages and disadvantages of each approach as well as future improvements.

## 3. Diabetes

### 3.1. Blood Glucose Homeostasis

**Figure 2:**
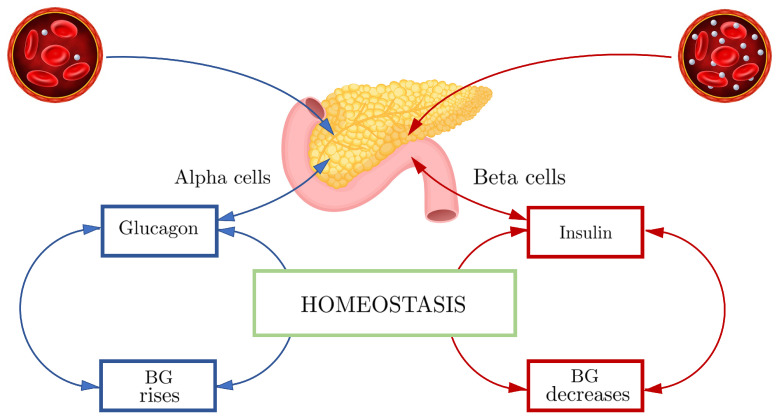
Blood glucose homeostasis process.

Glucose concentration inside the blood is naturally regulated by our body to maintain homeostasis (the tendency toward a relatively stable equilibrium between interdependent elements) [21]. In normal conditions, blood glucose is kept inside a narrow range. It should be less than 100 mg/dL after 8 h fasting and less than 140 mg/dL two hours after eating. During the day, its level is lower and, in a non-pathological subject, it stabilizes around 80 mg/dL [22]. Insulin and glucagon are the main actors of blood glucose regulation inside our body. They are two hormones secreted by the pancreatic islets (islets of Langerhans), the pancreas region that contains its endocrine cells. Inside the pancreatic cells, we can find, among other endocrine cells, the beta cells and the alpha cells that are responsible for secreting glucagon and insulin, respectively. Insulin is the only glucose-lowering hormone in our body. Glucagon, in contrast, has an increased-glucose effect. In physiological conditions, the release products of the beta cell inhibit the alpha cell function, while the release products of the alpha cells work as a stimulus for beta cells [23]. Due to its dimensions, glucose cannot pass through the cell membrane by simple diffusion. Insulin is able to lower glucose inside the blood by allowing its transport from the blood to the cells. The secretion of insulin is glucose dependent. When the blood glucose level rises (food intakes), more insulin is secreted, allowing the cells to utilize the glucose to generate energy. In opposition, when the blood glucose level decreases (exercises, insulin infusion, long fasting), more glucagon is secreted. Glucagon induces the liver to release its storage of glucose by converting the glycogen into glucose through the glycogenesis process. Glucagon also induces the liver and some muscles to generate glucose starting from lipids. Symptoms of diabetes are fast weight loss, abnormally high thirst, large amounts of urine production, fatigue and weakness, mood changes and blurred vision. Figure 2 shows a graphical representation of the glucose homeostasis.

### 3.2. Type 1 Diabetes

Type 1 diabetes (T1DM) is an autoimmune condition characterized by the lack of insulin production from the pancreas. It occurs when the immune system attacks and destroys the insulin-producing beta cells of the pancreas. In fact, beta cells loss is observable while alpha cells are preserved. However, the alpha cells function appears to be compromised too [24]. T1DM usually appears during childhood or adolescence but it can develop in adults too. People that are affected by T1DM have to take insulin through all their life to avoid complications.

### 3.3. Type 2 Diabetes

Type 2 diabetes (T2DM) is distinguished by a decreased sensibility to insulin. While in T1DM, there is no production of insulin due to the loss of beta cells, in T2DM, the insulin is produced but the secreted quantity is not enough to maintain homeostasis. In addition, the muscles and liver become insulin resistant. They do not respond anymore to the produced insulin. This condition, once again, generates high blood glucose concentrations. Type 2 diabetes is the most common around the world, and it mostly appears during adulthood [2]. It can be treated with regular physical exercises, healthy eating, weight loss and, in some cases, insulin therapy.

### 3.4. Gestational Diabetes

Gestational diabetes can happen at any stage of pregnancy, but is more common in the second or third trimester. It is not caused by a lack of insulin production but from the impossibility of correctly using the secreted insulin due to a hormone produced by the placenta. Glucose is stored in the blood instead of being absorbed by the cells. Increased thirst and more-frequent urination are possible symptoms, but most women do not notice any. Most of the time, it does not cause birth defects, and it can be easily managed with healthy eating and physical exercise. Women who have gestational diabetes are more likely to develop T2DM during their life [25].

### 3.5. Diabetes Complications

Diabetes complications can be correlated with hyper/hypoglycemia events. Hypoglycemia (blood glucose below 70 mg/dL) is often caused by diabetes treatments. Even though most of the time it is easily manageable by quickly getting the blood sugar back to physiological values by drinking or eating, when it frequently happens, it can be dangerous. Hyperglycemia is the condition in which the blood sugar concentration is above 180 to 200 mg/dL. When the body cannot take energy from glucose (due to the lack of insulin or insulin resistance), it converts the fat into energy with the production of waste products called ketones. Our body is not able to eliminate all the produced ketone leading to the life-threatening condition known as ketoacidosis (diabetic coma) [26]. However, the principal cause of death in diabetic subjects is represented by cardiovascular disease [27]. Macro-vascular manifestations include atherosclerosis and medial calcification [28,29]. Atherosclerosis is a specific type of arteriosclerosis. In atherosclerosis, the arterial walls narrow due to the formations of fat, cholesterol and other substance plaques. Although atherosclerosis is often considered a heart problem, it can affect arteries anywhere inside the body. Medial calcification is a condition characterized by the presence of diffuse calcium deposits along the medial layer of the arterial wall. Micro-vascular consequences are mainly represented by retinopathy and nephropathy [30]. Retinopathy is the most common vision impairment in diabetic subjects [31]. Elevated blood glucose leads to capillary occlusion, provoking the uncontrolled increase in local growth of new vessels in the retina. The dead capillary and the new ones can cause swellings, fluid leaks and retinal hemorrhages. When left untreated, it can lead to blindness. Diabetic nephropathy [32] is defined as the nephron’s inability to correctly filter impurities in the blood. This condition can lead to kidney failure and may be treated only with lifelong dialysis. Another complication that can appear in diabetic subjects is represented by neuropathy [33]. Peripheral nerves show issues as deficiencies in motor and sensory function. Autonomic peripheral neuropathy is the one type of diabetic neuropathy, and it affects the functionality of the organs innervated by the autonomous system (cardiovascular system, gastrointestinal system, pupillary system, etc.).

### 3.6. Conventional Tests for Diabetes Diagnosis

All the conventional methods for diabetes detection are invasive by nature. The glucose level is analyzed starting from a blood sample. Depending on the type of exam, the blood glucose level has to be maintained inside certain levels.

The fasting plasma glucose (FPG) blood test measures your blood glucose level at a single point in time. For the most reliable results, it is best to have this test in the morning, after at least 8 h fasting. A fasting blood sugar level of 99 mg/dL or lower is normal, 100 to 125 mg/dL indicates the presence of prediabetes, and 126 mg/dL or higher indicates the presence of diabetes. The oral glucose tolerance test (OGTT) measures blood glucose after an 8 h fast. It is used mostly to detect gestational diabetes. The blood is analyzed before and after the intake of a liquid containing a fixed amount of sugar. The blood is analyzed each hour following the intake, for two or three hours. High blood glucose levels at any two or more blood test times during the test results in a diabetes diagnosis. The OGTT is used not only with pregnant women to detect gestational diabetes, but also to detect type 2 diabetes. However, the OGTT is a more expensive and long test, and for those reasons, it is not frequently used. The casual plasma glucose test is another method of diagnosing diabetes. During the test, blood sugar is tested without regard to the time since the person’s last meal. A glucose level greater than 200 mg/dL may indicate diabetes. The hemoglobin A1c test (also called the glycated hemoglobin test or HbA1c) provides an average of the blood sugar control over a 6 to 12 week period. Hemoglobin is a protein contained in the red blood cells. It becomes glycated when it combines with glucose. The HbA1c test can be used to diagnose diabetes (if a value of equal to or greater than 6.5% is found) but also as a control to adjust the diabetes treatment (see Table 1).

### 3.7. Diabetes Treatments

Type 1 diabetes is mostly treated with manual insulin injections or insulin pumps. The blood glucose level has to be checked frequently in order to adjust the insulin treatment. Type 2 diabetes can be managed with changes in lifestyle, and monitoring of the blood sugar level, along with diabetes medications, insulin or both. In order to manage the pathology, diabetic subjects need to frequently check their glucose at home. For this purpose, several glucose meters have been commercialized. In order to be clinically validated, they need to comply with the medical device regulation. However, several different guidelines are proposed by different institutions [34]. A common approach to evaluate the performances of a glucose meter (or estimation model) is the Clarke error grid analysis. It was developed in 1987 to quantify the clinical accuracy of patient estimates of their current blood glucose as compared to the blood glucose value obtained in their meter [35]. The grid breaks down a scatterplot of a reference glucose meter and an evaluated glucose meter into five regions. Inside region A, there are those values within 20% of the reference sensor. Region B contains points that are outside of 20% but would not lead to inappropriate treatment. Inside region C, there are those points leading to unnecessary treatment. Points falling inside region D are considered a potentially dangerous failure to detect hypoglycemia or hyperglycemia. Finally, region E contains those points that would confuse the treatment of hypoglycemia for hyperglycemia and vice versa, leading to life-threatening errors.

## 4. PPG and ECG

### 4.1. The Cardiac Cycle

The cardiac cycle is a series of pressure changes that take place within the heart. Thanks to the presence of pacemaker cells inside the sinoatrial node, the heart is able to pump blood inside our body. The left part of the heart receives oxygenated blood from the lungs and pumps it inside the body. The right part receives deoxygenated blood from the body and pumps it toward the lungs to receive oxygen, thanks to the respiration. The cardiac cycle is mainly constituted by two different events, known as systolic and diastolic phases. The systolic phase is determined by the highest pressure inside the heart. From a medical point of view, it would be correct to speak about atrial systolic when the atrium contracts and pumps the blood inside the ventricle, and ventricular systolic when the ventricle contracts and pumps the blood either in the pulmonary circle or in the systemic circulation. The diastolic phase corresponds to the condition in which the atrium and ventricle are relaxed and the lowest pressure is detected inside the heart. Those pressure changes generated from the ventricular ejection are transmitted to the periphery of the systemic circulation, thanks to the different elasticity of the vessels, changing the volume of the blood inside them [36].

### 4.2. Electrocardiography

The electrocardiography records the electrical activity of the heart using electrodes placed on the skin. Its electrical activity is the consequence of the cardiac muscle depolarization followed by re-polarization during each cardiac cycle. It is largely employed in the medical field to investigate changes in heart dysfunction, such as arrhythmia and conductance issues [37].

**Figure 3:**
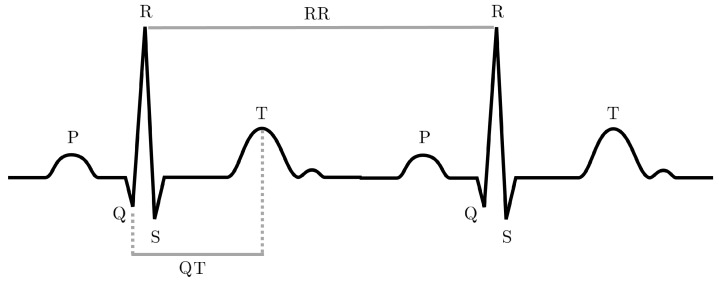
QRS complex in an ECG recording. RR: time between two consecutive R peaks (heart rate). QT: time between Q wave and T wave.

Since the registered signal is a difference of potential, the signal shows different wave shapes according to the derivation that is used. Generally, to compute ECG analysis, the second derivation is used. In this configuration that is registered by placing the electrodes on the wrists and on the left leg (arms and legs are considered as zones with equal potential), the QRS complex is observable in its well-known shape. The ECG signal is composed of a sequence of positive and negative deflections that reflect the electrical activity of the heart. The P wave is a small positive deflection that indicates atrial depolarization. The QRS complex appears as a small negative deflection followed by a positive peak provoked by the ventricular depolarization. T waves represent the repolarization of the ventricles. In Figure 3, a QRS complex and some of the most-used ECG features are shown. The ECG signal is often used to compute the HRV analysis. It can be implemented with several different approaches. While the time domain analysis is based on time-related parameters, the frequency domain analysis is focused on the parameters that can be computed based on the spectrum of the signal. Examples of time domain HRV features are the RR parameter (time between two adjacent R peaks), the SDRR parameter (standard deviation of RR interval) or SDNN (standard deviation of RR corrected interval). When computing a frequency analysis, the signal is decomposed into its components using mainly the FFT (fast Fourier transform) analysis [38]. The frequencies of interest are ultra low frequency ULF (<0.003 Hz), very low frequency VLF (0.0033–0.04 Hz), low frequency LF (0.04–0.15 Hz) and high frequency HF (0.15–0.40 Hz). In addition to these analyses, it is possible to compute the non-linear HRV analysis. Non-linear measurements index the unpredictability of a time series, which results from the complexity of the mechanisms that regulate HRV. Examples of non-linear analysis is the Poincaré plot from which S (area of the ellipse that best fits the data points), SD1 (ellipse width or short term HRV) and SD2 (ellipse length or long term HRV) result. Other parameters, such as approximate entropy (ApEn), sample entropy (SampEn) and detrended fluctuation (DFA), can be used to compute a non-linear HRV analysis [39].

### 4.3. Photopletysmography

**Figure 4:**
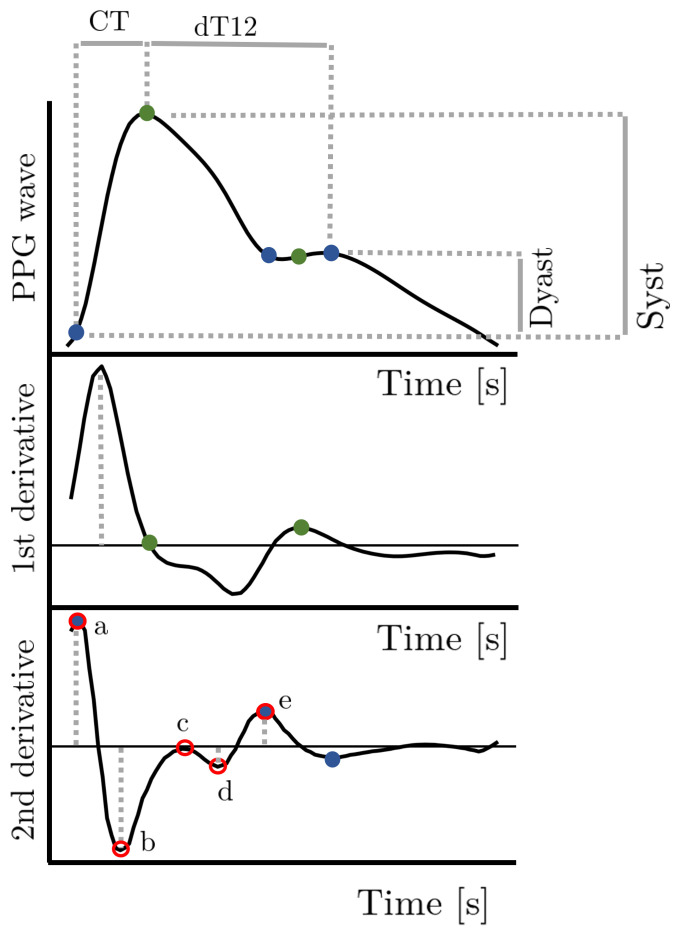
PPG signal and its first and second derivatives. Green points show the fiducial points identified through the first derivative analysis. Blue points show the fiducial points identified through the second derivative analysis. CT: Crest time. dT12: time between systolic and diastolic peaks. Dyast: diastolic peak amplitude. Syst: systolic peak amplitude. a, b, c, d, e: first five maxima and minima of the second derivative.

PPG is a low-cost, non-invasive and user-friendly technique used to detect blood volume changes in the micro vascular tissue bed through the skin. In fact, because the skin is so richly perfused, is it easy to detect the pulsatile component of the cardiac cycle using the PPG. A photoplethysmograph is composed by a light emitting diode (LED) and a photodetector (PD). The light emitted from the diode is reflected (or transmitted) through the tissues and the arteries and arterioles and then detected by the photodetector [40]. The wave is composed of two components: forward and backward waves. The forward wave, also called systolic component or direct wave, is related to the systolic ejection. The backward component, also called diastolic component or reflected wave, is provoked by the return of the blood from the peripheral circulation to the center due to arterial elasticity [41]. Traditionally, it was used in clinical fields to assess oxygen saturation. With the growing interest in tele-home healthcare, PPG embedded in wearable sensors have started to be used to detect multiple cardiovascular diseases (CVD) and indexes, such as atrial fibrillation, heart rate, heart rate variability and blood glucose level. The photoplethysmographic waveform depends on several factors such as the wavelength emitted light, on the anatomical recorded site, on tissue reflectance, on blood volume, cardiac cycle phase, aging and the possible presence of pathologies [42,43,44]. The finger, earlobe and wrist appear to be the best measurement sites with, respectively, 95%, 81% and 86% analyzable waveforms due to the rich arterial supply [45]. Thanks to their convenience to affix sensors, the earlobe and finger became the most used anatomical sites for PPG clinic measurements. However, lately, several studies proposed innovative solutions with wrist PPG, thanks to the innovation of the wrist device technology [46]. An example of PPG signal and the most common extracted features is shown in Figure 4. Some parameters that characterize the HRV analysis can also be computed using PPG signals by using the systolic peak as the R peak in the ECG signal.

## 5. Results and Discussion

**Figure 5:**
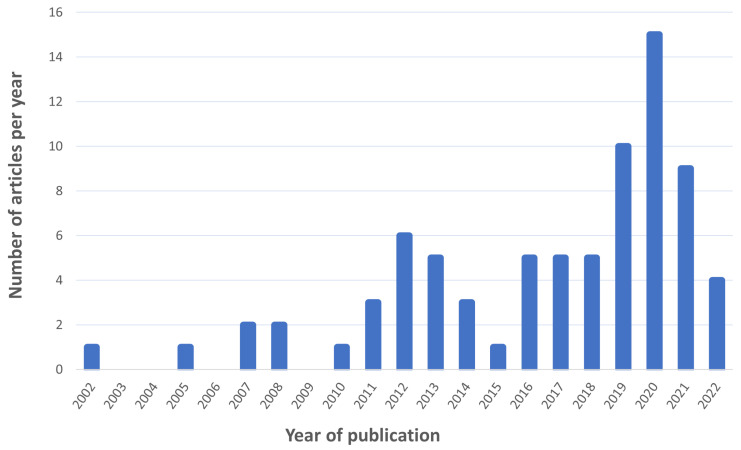
The number of included articles published per year.

A total of 227 articles were downloaded. We also completed a search of the already published reviews on this topic. Only two reviews about diabetes care and ECG/PPG signals were found. Eighty articles were excluded because they are out of context. We fully analyzed 147 articles in total. Sixty-nine articles were further excluded due to the presence of duplicates or lack of information. The articles were finally classified into three main categories: diabetes detection, blood glucose estimation and diabetes complications. A total of 31 articles for the diabetes detection, 41 articles about blood glucose detection and 7 about diabetes complications were found. We classified the included studies into subclasses based on the proposed approach: traditional, machine learning and deep learning. The articles found concerning diabetes detection are composed of 10 traditional approaches, 15 machine learning and 6 deep learning approaches. A total of 5 traditional approaches articles, 21 machine learning and 15 deep learning articles were found for the blood glucose estimation. Concerning the diabetes complications, we found and included five traditional, one machine learning and one deep learning approach. Figure 5 shows the number of included articles published per year.

As described in Section 3, diabetes can affect the cardiovascular system in many ways. Even though the directionality of the relation between diabetes and cardiovascular diseases is still unknown, it is well accepted that diabetes and increased arterial stiffness are linked [47]. It is also confirmed that blood viscosity increases with blood glucose [48]. HRV showed to be lower, in all age groups, in diabetic patients with respect to healthy subjects [49]. In addition, modification in ECG recordings was observed in the presence of diabetes [50]. Arterial walls stiffness, blood viscosity and modifications of the heart polarization/depolarization directly affect PPG wave shapes. Thanks to all these factors, diabetes can be potentially detected by analyzing the PPG and ECG signals.

**Figure 6:**
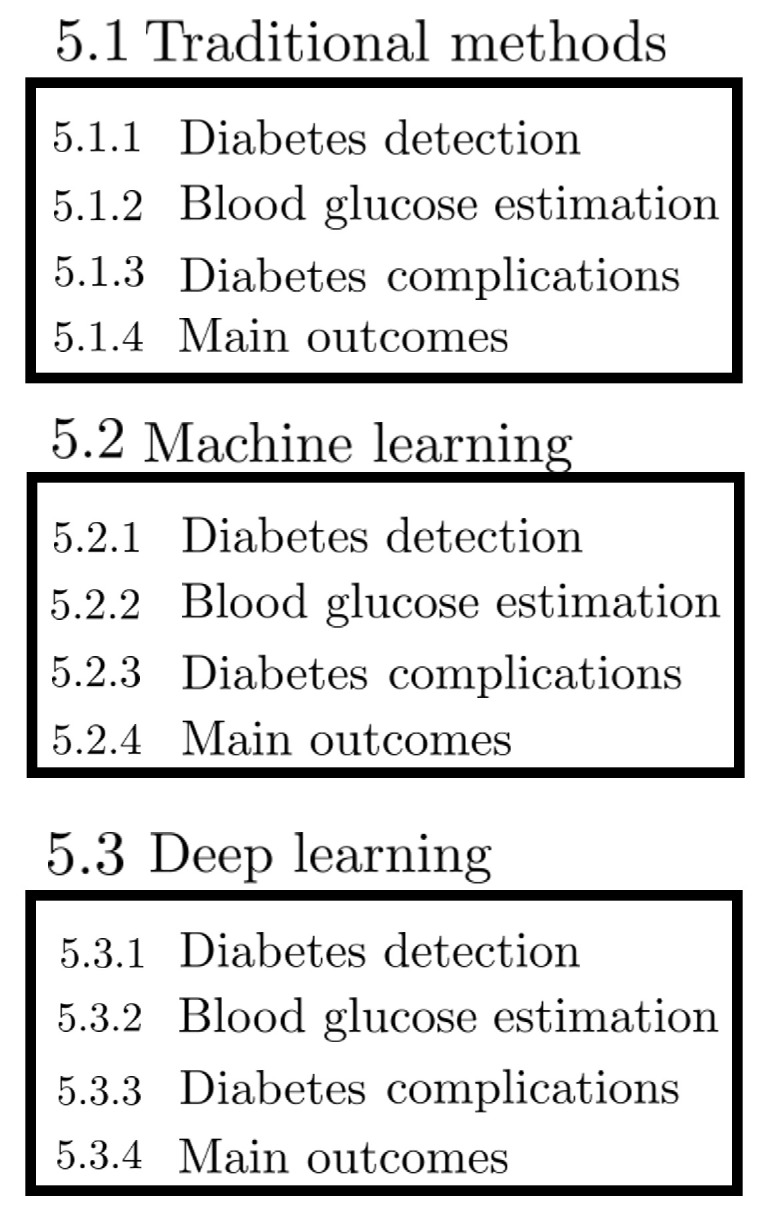
Graphic representation of this section organization.

In this section, we present a summary of the published studies about diabetes detection, blood glucose estimation and diabetes related complications detection through the analysis of PPG and ECG signals. First we present the traditional approaches, then the machine learning ones. Finally, we cover the deep learning approaches. The studies are collected into separated tables based on their main objective (diabetes detection, blood glucose estimation and diabetes complication). The most significant ones are equally presented in the text. Figure 6 shows a graphical representation of the organization of this section.

### 5.1. Traditional Methods

In the traditional methods category, we inserted statistical analysis and mathematical models that do not belong to the machine or deep learning domain. While very few mathematical models have been implemented in diabetes care with PPG and ECG, several articles assess the PPG and/or ECG based feature significance among diabetic and non diabetic subjects. In this section, we present the traditional approaches to detect diabetes, estimate blood glucose and detect diabetes complications through PPG and ECG analyses.

#### 5.1.1. Diabetes Detection with Traditional Methods

The presented studies detect diabetes mostly by computing the HRV analysis or by assessing differences among ECG and/or ECG features. Faust et al. [51] compare linear and non-linear HRV ECG methods applied to 15 diabetic and 15 non-diabetic subjects. Non-linear parameters were found to be clinically and statistically significant among the two groups. Mean values for SD2, CD, ApEn and SampEn are higher in normal with respect to diabetic subjects. Higher values for normal subjects indicate that normal subjects have higher HRV. These findings are in line with current medical understanding, because a diabetic subject is, at least in a statistical sense, less active than a healthy person. This result was confirmed also in the study presented by Seyd et al. [52]. They computed time and frequency HRV ECG analyses between 16 diabetic and 16 normal subjects. LF % power and HF power are shown to be statistically higher in healthy subjects with respect to diabetic. Another interesting approach was presented by Haryadi et al. [53]. They applied the multiscale Poincarè analysis to the PPG amplitude to differentiate healthy subjects, healthy diabetic subjects and unhealthy diabetic ones (16, 18 and 18, respectively).

The results highlight the feasibility of MSP (multi-scale Pointcaré) application in diabetes detection analysis for computational time reduction and sensitivity enhancement. Table 2 presents the available works on detecting diabetes using PPG and ECG with traditional approaches. None of the presented studies used public datasets.

#### 5.1.2. Blood Glucose Estimation with Traditional Methods

Table 3 presents an overview of blood glucose level estimation through ECG and PPG signals with traditional approaches.

The articles are mainly focused on hypo and hyperglycemia events detection. In fact, serious hypoglycemia can lead to the variation of the ECG parameters, such as QT segment length and HRV variations. Singh et al. [61] computed linear HRV analysis on 1919 diabetic subjects. SDNN, LF and HF were inversely related to plasma glucose levels (*p* < 0.0001). After adjusting for covariates, such as age, sex and body mass index, the LF power and LF/HF ratio were lower in DM subjects than in those with normal fasting glucose (*p* < 0.005). However not only hypoglycemia modifies ECG parameters. Ngyuen et al. [64] showed that ECG significant modifications are observable also during hyperglycemia events. The study involved five subjects, who experienced both hyperglycemia and hypoglycemia events. The hypoglycemic state had significant increase in heart rate, QTc (corrected ECG QT interval), RTc (corrected ECG RT interval) and TpTeC (ECG T wave peak-to-end interval) (all with *p* < 0.0001). During hyperglycemic states, there were significant increases in PR (*p* < 0.0001), decreases in QTc (*p* < 0.0001), RTC (*p* = 0.001) and TpTeC (*p* = 0.004). In all the three groups, HR (r = −0.466) was moderately related to BGLs (blood glucose levels), while QTc, PR, RTc, TpTeC had strong relations with BGLs (r = −0.712, 0.783, −0.781, −0.586, respectively, all with *p* < 0.0001). None of the presented studies employed PPG signals. To our knowledge, there are no published articles on estimating blood glucose level from PPG uniquely with traditional approaches.

#### 5.1.3. Diabetes Complications with Traditional Methods

An overview of the published studies on detection diabetes complications with ECG and/or PPG signals with traditional approaches is shown in Table 4.

The presented studies are focused on diabetic peripheral neuropathy (DPN) [71] and on cardiac autonomic neuropathy (CAN) [72]. Al-Hazimi et al. [68] used ECG signals to compute linear HRV analysis in order to assess differences between 10 healthy subjects, 10 diabetic and 10 diabetic with DPN. While linear HRV analysis is able to distinguish between healthy and diabetic, as proven in the previous section, no significant differences were found between diabetic subjects and diabetic subjects with DPN. However, other types of ECG analysis seem to provide promising results. Wei et al. [67] demonstrated that the percussion entropy index (PEI) computed from synchronized ECG and PPG signals is a DPN predictor. In this study, 37 healthy subjects and 85 diabetic without DPN were enrolled to collect ECG and PPG signals. The subjects were followed for 6 years after the baseline measures. Out of 85 diabetic patients, 25 developed DPN. Among all the computed parameters, PEI showed significant differences among the three groups (*p* < 0.001). Cornforth et al. [69] demonstrated that Renyi entropy shows significant differences between diabetic with and without CAN. Renyi entropy seems to be able to detect between noCAN, early CAN and definitive CAN with high significance level (*p* < 0.001). The only study that assessed diabetes complication uniquely from PPG signal is presented by Kim et al. [66]. Since the control group is composed of healthy subjects, it is difficult to affirm if the proposed parameters were able to detect and isolate the diabetes complication or only succeeded in detecting diabetes.

#### 5.1.4. Traditional Approaches: Main Outcomes

Most of the proposed studies focused on assessing significant differences in PPG and/or ECG derived parameters among different subjects. HRV-derived parameters demonstrated to be statistically different in subjects with diabetes with or without hyper/hypoglycemia events with respect to healthy subjects. However, no significant differences were found in diabetes complication detection. Most articles focused on ECG rather than PPG analysis. No studies were found for blood glucose estimation with traditional approaches with ECG and/or PPG signals. Overall, the traditional approaches showed good results in detecting parameters modifications due to diabetes. One of their main advantages is represented by the low computational complexity. However, they are hardly applicable in real-time analysis and require a quality assessment step. In fact, when dealing with feature extraction, poor quality waves can heavily affect the results reliability. In addition, they are always computed from the entire dataset. This implication results in no separated test analysis. A parameter that resulted in being significantly different in a population could be not significant when applied to another group of subjects.

### 5.2. Machine Learning

Before the advent of deep learning, biosignals were mainly analyzed with machine learning (ML) approaches. The ML approaches require less data and they are more interpretable, compared to the deep learning ones. However, they require, as for traditional approaches, the feature extraction step. In this section, we present the machine learning approaches to detect diabetes, estimate blood glucose and detect diabetes complications through PPG and ECG analysis.

#### 5.2.1. Diabetes Detection with Machine Learning

An interesting article about diabetes detection was proposed by Monte-Moreno et al. [73]. In this study, the cepstral analysis was computed over PPG signals from 830 diabetic and 340 healthy subjects. In signal processing, the cepstral analysis is defined as the inverse Fourier transform (IFT) of the logarithm of the estimated signal spectrum. Mathematically, it deals with the problem of the deconvolution of signals in the frequency space. It can be used to perform HRV analysis. In this study, the several parameters extracted from the cepstral analysis were used in combination with frequency domain PPG features and demographic data. Several machine learning models were trained to distinguish between diabetic and healthy subjects. The best performance was obtained by a random forest approach, scoring 64% of specificity and 65% of sensitivity. The authors stated that those results are comparable with the hemoglobin A1c HbA1c test. Another HRV based analysis was proposed by Chu et al. [74]. In this case, a classical frequency HRV analysis was computed from 1228 diabetic and 1310 healthy subjects. Two different logistic regression models were trained: one model was fitted with the HRV features and the subjects’ waist circumference, the other was fitted only with HRV features. The models were trained to predict over 5 diabetes risk levels. The best performance was reached from a hybrid model composed by both previous models (with and without waist circumference). This model scores an accuracy of 90%, a specificity of 88% and a sensitivity of 92.5%. A different approach was proposed by Rajendra Acharya et al. [75] who performed discrete wavelet transform (DWT) decomposition ECG signals in order to distinguish between 15 diabetic and 15 healthy subjects. They extracted the energy, sample entropy, approximation entropy, kurtosis and skewness features at various detailed coefficient levels of the DWT. These features were then used to train several methods of machine learning. Decision tree reached an accuracy of 92.02%, sensitivity of 92.59% and specificity of 91.46%. Table 5 presents the available works on detecting diabetes using PPG and ECG with machine learning approaches.

#### 5.2.2. Blood Glucose Estimation with Machine Learning

Blood glucose estimation and prediction can be used to predict hypo/hyperglycemia events but also to adjust the insulin dose to be injected in order to maintain the homeostasis. Due to this important role, as for conventional glucose meters, blood glucose estimation models need to satisfy some strict criteria in order to be considered clinically validated. To this purpose, the Clarke grid error analysis is often proposed. In his study, Monte-Moreno [89] trained several machine learning models to estimate blood glucose levels and blood pressure from 410 subjects (339 healthy and 79 diabetics). Time and frequency PPG features and physiological data, such as BMI and age, compose the 21 features vector used to fit the models. A random forest based model scored the best performance in terms of stability and R^2^. The results show that 87.7% of points were in region A and 10.3% of the data went to region B. Since none of the estimated points fall into regions C, D or E, this approach is validated according to the Clarke error grid analysis. Zhang et al. [90] estimated three levels of blood glucose: normal, borderline and warning. The subjects (30 diabetic subjects and 50 healthy subjects) were asked to collect video PPG. Once the PPG signal is extracted from the video, a Gaussian fitting is applied. Two Gaussian functions are fitted in order to model the forward and backward components of the PPG wave. A total of 28 features, composed of time and frequency PPG features and Gaussian fitted curve based features, were used to train several machine learning models. A support vector machine based model scored the best performance, reaching an accuracy of 81.5% (specificity 83.2% and sensitivity 80%). While PPG signals are mostly used to estimate the exact level of glucose, ECG signals are primarily used to detect hypoglycemic events. Cichosz et al. [91] showed that ECG can enhance the prediction of hypoglycemia events. They compared the performance of a general continuous glucose meter (CGM) with respect to their system that combines CGM information with the HRV ECG analysis. Their system appeared to have a better specificity (99% vs. 93%), higher ROC (receiver operating characteristic) AUC (area under the curve) (0.98 vs. 0.96) and a larger lead time, 22 versus 14 min, while the sensitivity was slightly lower (79% vs. 81%). Ling et al. [92] developed a hybrid particle-swarm-optimization-based fuzzy reasoning to detect hypoglycemic events during the night. ECG was recorded overnight from 16 children with T1DM. The fuzzy reasoning model (FRM) is used to correlate HR and QTc to hypoglycemia. The model is able to detect advanced hypoglycemic episodes (sensitivity is 85.7% and specificity is 79.87%) and hypoglycemic episodes (sensitivity is 80% and specificity is 55.1%). Table 6 presents the available works on blood glucose level estimation using PPG and ECG with machine learning approaches.

#### 5.2.3. Diabetes Complications with Machine Learning

Concerning the diabetes complications detection with machine learning approaches, only one article was found. Jelinek et al. [110] proposed a graph-based machine learning system (GBMLS) to detect CAN. A total of 21 subjects with CAN took part in this study. The model was able to detect the severe neuropathy with a sensitivity of 89% and a specificity of 98%. Since the neuropathy is not a systematic consequence of diabetes, the control group should be composed by diabetic subjects without complications. However, the study does not clearly report this information. In such a case, the model performance would refer to its capability in detecting diabetes rather than its capability to differentiate between a diabetic subject and a diabetic subject with neuropathy. Table 7 contains the details of the cited article.

#### 5.2.4. Machine Learning: Main Outcomes

Also with machine learning, as for traditional approaches, HRV-derived parameters are often employed. Those features are fitted into a variety of machine learning methods in order to detect diabetes or to predict blood glucose level. ECG and PPG signals are equally employed for this purpose. Support vector machine or regression, decision tree and random forest are the most common approaches. Regarding the detection of diabetes complications, further studies need to be done to better investigate the topic. Overall, promising results are shown in the presented studies. However, much attention should be put on the dataset processing step. A rigorous separation between train test and validation is needed in order to obtain reliable results. In some cases, once the feature extraction is completed, the dataset is automatically split into train, validation and test. In this case, it is very likely that features belonging to the same subject are contained in more than one partition. The splitting process should be computed as the first step and based on the available subjects. Another limitation that can be observed is the lack of results standardization. The variety of available methods to analyze the performance of the proposed methods makes the comparison challenging.

### 5.3. Deep Learning

Recently, deep learning methods were largely employed in biomedical signal processing [111]. Their main limitations are represented by the difficulty to interpret the model and by the amount of data that they require. However, their use grew fast thanks to the possibility of avoiding the feature extraction process that, when working with real signals, can be very challenging. In this section, we present the deep learning approaches to detect diabetes, estimate blood glucose and detect diabetes complications through PPG and ECG analyses.

#### 5.3.1. Diabetes Detection with Deep Learning

The great advantage of deep learning is the possibility to employ raw signals without extracting significant features. Due to this reason, convolutional neural networks (CNNs) are largely employed in PPG and ECG analyses. In fact, their main characteristic is the capability of auto-extracting features using the convolution filters between one layer and the next one. The convolution process reduces the size of the input, preserving its important characteristics. Avram et al. [112] proposed a CNN-based model to detect diabetes from raw PPG signals. The proposed model is composed of 18 CNN layers. Their dataset is composed of 53,870 individuals that were asked to collect PPG signals from a smartphone camera. The network reached a specificity of 65.4%, a sensitivity of 75% and an AUC of 0.77 on average over three test datasets. In addition to the CNN model, they built a logistic regression based model. The model takes as input the CNN output and some physiological data, such as body mass index (BMI), comorbidities, age and gender. The best result was obtained when the CNN score and comorbidities were used together (AUC = 0.83). Comparable results, with different approaches, were presented by Wang et al. [113] and Srinivasan et al. [114]. Wang et al. [113] utilized ECG images to train a 2D-CNN model to distinguish between normal ECG records and prediabetic ones. The train dataset is composed of 1750 normal samples and 501 prediabetes samples. The actual number of analyzed subjects is not clearly reported. The study shows that the model performed better when trained and tested on datasets that contain similar BMI subjects with respect to a mixed one. The best performance was obtained from normal-weight subjects (BMI < 25), scoring 84%, 90% and 0.83 as sensitivity, specificity and AUC, respectively. Another two-dimension approach was proposed by Srinivasan et al. [114]. The presented model is trained over PPG scalogram images computed from 467 normal subjects and 341 diabetic subjects PPG. Also in this case, several combinations of inputs were tested. The best performance was reached when the PPG scalogram was used as input in combination with age, gender and presence of hypertension (sensitivity: 76.6%, specificity 76.1%, AUC: 0.83). Table 8 presents an overview of the available works on detecting diabetes using PPG and ECG with deep learning approaches. It is important to notice that a very high value of accuracy, specificity or sensitivity could be the result of a not complete separation between train/validation and test set.

#### 5.3.2. Blood Glucose Estimation with Deep Learning

In the blood glucose domain, few articles fully exploited the deep learning advantages of being capable of dealing directly with raw signals. Most of the proposed articles focused on extracting features clinically related to blood glucose variations. Habbu et al. [119] proposed a feature-based approach to estimate the blood glucose level. The dataset was composed of 378 healthy subjects and 233 diabetic. In this study, the authors explored two approaches. Eight cepstral coefficients (CC), their standard deviation (SD) and variation were computed on two different parts of the signal. First a frame approach was exploited using a 1 s PPG window. In addition to that, a single pulse approach was studied too. Two MLP (multi layer perceptron) models were trained to assess the differences in the performance of the window-based approach with respect to the single pulse approach. The single pulse approach overcame the window approach, obtaining a better precision of estimation (85.2% A region, 13.6% B region, 1.2% C region with respect to 77.8% A region, 19% B region, 1% C region, 1.6% D region and 0.6% D region). However, this approach does not meet the requirements from the Clarke error grid analysis. Cordeiro et al. [120] trained several machine and deep learning models to detect hypoglycemia. He computed innovative HRV features from ECG signals belonging to 1119 subjects. In addition to time HRV features, the authors also investigated the importance of the ECG waves slope. The model was trained over 9 ECG feature length and slope (PQ, PR, PS, QR, QT, RS and RT). The model that outperformed all the others is a MLP-based model. It was able to detect hypoglycemia events with a specificity of 85% and a sensitivity of 87.5%. This result, also taking into consideration the dataset size, overcomes the other studies’ results. As a no-features based approach, Porumb et al. [121] used raw ECG signals to detect hypoglycemia. The dataset is composed of 5 healthy subjects, and the study aim is to provide a personalized approach by training one model for each subject. To this purpose, the subjects were monitored 24 h for 14 days. Also in this case, two different approaches were tested. The first consists in training a CNN network with single ECG pulses and activity value. The second one consists of a CNN + RNN network that takes as input 200 consecutive ECG beats and activity values. The combination of CNN and RNN overcame the CNN performance, scoring on average 86%, 81% and 82% of sensitivity, specificity and accuracy, respectively.

Table 9 presents the available works on blood glucose level estimation using PPG and ECG with deep learning approaches.

#### 5.3.3. Diabetes Complications with Deep Learning

From our literature search, only one article assessed diabetes complications through deep learning approaches. Alkhodari et al. [133] proposed a study to detect diabetes complications. The dataset is composed of 25 healthy and 75 diabetic subjects. The diabetic group is further composed of diabetic with different complications, such as CAN, DPN, nephropathy and retinopathy. ECG HRV parameters (linear and nonlinear) were used to test different combinations of datasets (i.e., healthy vs. diabetic, diabetic vs. diabetic with complications, and diabetic vs. diabetic with CAN). Among all the tested models, CNN scored the best results in distinguishing diabetic healthy subjects from diabetic subjects with micro vascular complications (all of them). The sub dataset is composed of 4 diabetic without complications and 66 subjects with complications. The model scored an accuracy of 98.5%, sensitivity of 100% and specificity of 97%. Table 10 contains the details of the cited article.

#### 5.3.4. Deep Learning: Main Outcomes

The main advantage of deep learning approaches is the possibility of avoiding the feature extraction step that can be very challenging when real ECG and/or PPG signals are employed. This capability is mostly employed in diabetes detection, while for blood glucose estimation and diabetes-related complications, features-based approaches are still preferred. Among the possible features, HRV-derived features are the most common ones. Also in this case, as for machine learning, particular attention should be put in correctly splitting the dataset to avoid biased results. Although it is well accepted that deep learning requires a large amount of data to produce reliable results, the majority of the studies used small datasets. This fact could be justified by the low complexity of the proposed models. In fact, as shown by Brigato et al. [134], low complexity models perform comparably well or better than high complexity models when dealing with small datasets. However, significant work has to be done in order to further study the models’ interpretability and clinical validation. It is a crucial requirement that needs to be satisfied to employ deep learning models in a clinical environment.

## 6. Conclusions

Diabetes is a worldwide concern. Conventional methods for diabetes testing and managing, such as CGM and HbA1c, are invasive and can be expensive. Several studies have been conducted to detect and manage diabetes with the help of big data. Food intake, steps count, voice analysis and EHR are only some of the data that have been exploited for this purpose. However, it is important to highlight that the first cause of death in diabetic patients is cardiovascular disease [135]. Armed with this knowledge, several researchers recently approached the diabetes care field through ECG and PPG analyses as shown in this paper review. Several different approaches were proposed from the traditional to the deep learning ones. As traditional approaches, many PPG and ECG parameters were analyzed in order to detect significant variations that could define the presence of the pathology or a certain complication within a dataset. However, sometimes, simple approaches, such as linear correlation, cannot explain the complex relation among the data. For this purpose, often, the very same parameters are analyzed with machine learning approaches. Nonetheless, both traditional and machine learning approaches require the feature extraction step to be done. The extraction of fiducial points on real-life signals can be very challenging. Besides the difficulty of generalizing a feature extraction algorithm that could work on the very different kinds of waveforms, there is always the necessity of computing a quality assessment. In fact, no feature extraction algorithm can work if the input signal is corrupted. The deep learning advent helped the researcher to compute analysis of a very large amount of data, avoiding the feature extraction step. The downside of deep learning approaches is that they are not easily interpretable. In a clinical environment, this can be an issue since the knowledge of why and how a pathology was detected is fundamental in order to validate the diagnosis. Another limitation is the lack of standardization of the results. In fact, to this day, each study can propose any error result analysis. This heterogeneity makes it difficult to quantify the differences among the studies (in terms of results). In addition, the lack of information about the used dataset makes the reproducibility of the study very hard. Excluding the case in which a personalized model approach is proposed, much attention should be put in not using features or signals belonging to the very same patient in both the train and test. Not separating the subjects could lead to misleading results. In conclusion, PPG and ECG analyses to detect and manage diabetes is very promising.

To summarize, the main areas that need to be better explored are as follows:

- Data processing standardization.
  – In all the proposed approaches (traditional methods, machine learning and deep learning ones) signal processing and features extraction is a very important step. The authors should provide the maximum of details to allow reproducibility.
  – When testing the model is necessary (as for ML and DL), the splitting process should be performed with much attention to avoid biased results.
- Standardization of performance analysis.
  – Performance is analyzed with a variety of parameters. The wide spectrum of performance parameters allows researchers to highlight different advantages and limitations of the proposed method. Future studies should be focused on creating guidelines on performance analysis to allow cross analysis.
- Clinical validation.
  – To our knowledge, none of the proposed methods have been clinically validated. ECG and PPG analyses for diabetes care is still an open research topic. However, to achieve the objective of employing these analyses in clinical environments, clinical validation is a step that cannot be skipped.

## Figures and Tables

**Table 1:** Diabetes test values for diabetes and prediabetes.

Diabetes Test	Description	Pre Diabetes	Diabetes
Fasting plasma glucose	After 8 h fasting	100–125 mg/dL	>126 mg/dL
Casual Plasma Glucose	Any time	None	>200 mg/dL
Oral Glucose Tolerance	Fasting and every hour for 2 or 3 h	140–199 mg/dL	>200 mg/dL
Hemoglobin A1c	Any time	5.7–6.4%	>6.5%

**Table 2:** Traditional methods for diabetes detection.

Reference	Objective	Data Type ^a^	Approach	Feature	Main Outcome
Buchs et al. [54]	Healthy vs. Diabetic	PPG [10 min] 68[33/35] In-house	Right-left correlation	Amplitude, baseline variation and period	Lower correlation in diabetic subjects
Seyd et al. [52]	Healthy vs. Diabetic	ECG [1 h] 32[16/16] In-house	Statistical analysis	HRV	LF % power **, HF power ** lower in diabetic subjects
Usman et al. [55]	Healthy diabetic vs. Unhealthy diabetic	PPG [90 s] 56 [30/26] In-house	Statistical analysis	AUC	AUC *
Faust et al. [51]	Healthy vs. Diabetic	ECG [1h] 30 [15/15]In-house	Linear and non linear analysis	HRV	CD ***, ApEn ***, SampEn *** and recurrence plot properties ***
Wu et al. [56]	Healthy vs. Diabetic	PPG - ECG [30 min] 51 [27/24] In-house	Multi scale Cross-approximate Entropy analysis	HRV	MC-ApEnLS **
Pilt et al. [57]	Healthy vs. Diabetic	PPG [-] 44 [24/20] In-house	Statistical analysis	PPG Augmentation Index	PPGAI ***
Haryadi et al. [58]	Healthy vs. Diabetic vs. Unhealthy diabetic	PPG [1000 pulses] 52 [16/18/18] In-house	Multi scale poincaré analysis	Amplitude	SSR **
Hsu et al. [59]	Healthy vs. Diabetic	PPG [30 min]14 [48/46] In-house	Statistical analysis	CT, CTR, PWV	CTR **
Usman et al. [60]	Healthy diabetic vs. Unhealthy diabetic	PPG [90 s] 101 [53/48] In-house	Statistical analysis	Signal and 2nd derivative features	PPG slope angles **
Haryadi et al. [53]	Healthy vs. Diabetic	PPG [1000-500-250-100 pulses] 64 [34/30] In-house	Multi scale poincaré analysis	Amplitude	MSPI detect with higher sensibility wtr to the multiple temporal scale index and the single scale index.

Traditional methods for diabetes detection. ^a^: type of signal [signal length], total number of subjects [subjects for each class], type of database. CD: correlation dimension. ApEn: approximate entropy. SampEn: sample entropy. REC: recurrence plot. CF: complex fluctuation. SSR: ration between long and short variations. LF: low frequency. HF: high frequency. MC-ApEnLS: multiscale cross-approximate entropy in large scale. CT: crest time. AUC: area under the curve. CTR: crest time ratio. PWV: pulse wave velocity. * *p* < 0.05, ** *p* < 0.01, *** *p* < 0.001.

**Table 3:** Traditional methods for blood glucose estimation.

Reference	Objective	Data Type ^a^	Approach	Feature	Main Outcome
Singh et al. [61]	Hypoglycemia detection (T1DM)	ECG [2 h] 1919 [1779/140] In-house	Linear analysis	HRV	SDNN, LF, HF ***diminished LF/HF, HF **
Harris et al. [62]	Hypoglycemia detection (T1DM)	ECG [night] 52 [20/32] In-house	Statistical analysis	QT, QTc	4 out of 6 events correctly detected. QTc variaed more in diabetic subjects
Laitinen et al. [63]	Hypoglycemia detection	ECG [5 min] 18 In-house	Statistical analysis	PR, QT, QTc	PR decreased ** QTc increased ***
Nguyen et al. [64]	Hypoglycemia and hyperglycemiadetection (T1DM)	ECG [night] 5 In-house	Statistical analysis	HR, QTc, PR, RT, TpTe	Hypoglycemia: increased QTc, RTc, TpTe (***) Hyperglycemia: increased PR *** decreased QTc, RTc (***)
Amanipour et al. [65]	Hypoglycemia detection (T1DM)	ECG [1 h] 1 In-house	Linear analysis	HRV	LF/HF inversely correlated with BG

Traditional methods for blood glucose detection. ^a^: type of signal [signal length], total number of subjects [subjects for each class], type of database. SDNN: standard deviation of normal intervals. LF: low frequency. HF: high frequency. QT: ECG Q to T wave interval. QTc: corrected QT interval. PR: ECG P to T wave interval. RT: ECG R to T wave interva. RTc: RT corrected. HR: heart rate. TpTe: ECG T wave peak-to-end interval. ** *p* < 0.01, *** *p* < 0.001.

**Table 4:** Traditional methods for diabetes complications.

Reference	Objective	Data Type ^a^	Approach	Feature	Main Outcome
Kim et al. [66]	Healthy vs. Diabetic with DPN	PPG [30 s] 114 [64/50] In-house	Statistical analysis	Finger to toe ratio	Sensitivity: 98% Specificity: 92.2%
Wei et al. [67]	Healthy vs. Diabetic without DPN	PPG, ECG [1000 pulses] 122 [37/85] In-house	Percussion Entropy Analysis	PEI, MEI, LHR	PEI *** as indicator of future neuropathy
Al-Hazimi [68]	Healthy vs. Diabetic without DPN vs. Diabetic with DPN	ECG [24 h] 30 [10/10/10] In-house	Linear analysis	HRV	No significant difference found in detecting neuropathy
Cornforth et al. [69]	Diabetic vs. Diabetic with CAN	ECG [20 min] 149 [71/78] In-house	Multi scale Renyi entropy	Renyi Entropy	Renyi entropy ***
Imam et al. [70]	Diabetic vs. Diabetic with CAN	ECG [20 min] 80 [40/40] In-house	Bivariate and trivariate ARMA models	QT, RR, EDR	EDR model based was able to detect among the groups

Traditional methods for diabetes complications detection. ^a^: type of signal [signal length], total number of subjects [subjects for each class], type of database. PEI: percussion entropy index. MEI: multiscale entropy index. LHR: low high frequency ratio. QT: ECG Q to T wave interval. RR: ECG R to R wave interval. EDR: ECG derived respiration. *** *p* < 0.001.

**Table 5:** Machine learning methods for diabetes detection.

Reference	Objective	Data Type ^a^	Approach	Feature	Main Outcome
Amiri et al. [76]	Healthy vs. Diabetic	PPG [12 min] 30 [14/16] In-house	ARMA + SVM	Averaged ARMA model	Accuracy: 80% Sensitivity: 78.6% Specificity: 81%
Keikhosravi et al. [77]	Healthy vs. Diabetic	PPG [12 min] 46 [23/23] In-house	Bayesian classifier	SVD	Accuracy: 93.5% Sensitivity: 100% Specificity: 87%
Acharya et al. [78]	Healthy vs. Diabetic	ECG [2 s] 30 [15/15] In-house	AdaBoost	Signal features	Accuracy: 90% Sensitivity: 92.5% Specificity: 88.7%
Acharya et al. [79]	Healthy vs. Diabetic	ECG [1 h] 30 [15/15] In-house	AdaBoost	Non-linear HRV	Accuracy: 86% Sensitivity: 87.5% Specificity: 84.6%
Jian et al. [80]	Healthy vs. Diabetic	ECG [1 h] 30 [15/15] In-house	SVM	HRV (HOS)	Accuracy: 80% Sensitivity: 70.1% Specificity: 89.2%
Acharya et al. [75]	Healthy vs. Diabetic	ECG [1 h] 30 [15/15] In-house	Decision Tree	DWT features from HR signal	Accuracy: 92% Sensitivity: 92.6% Specificity: 91.5%
Monte-Moreno et al. [73]	Healthy vs. Diabetic	PPG [1 min] 1170 [340/830] In-house	RF, Gradient Boost	Signal features + physio data	Accuracy: 70% Sensitivity: 80% Specificity: 48%
Pachori et al. [81]	Healthy vs. Diabetic	ECG [1 h] 30 [15/15] In-house	LS-SVM	R-R IMFs parameters	Accuracy: 95.63% Sensitivity: 97.5% Specificity: 93.7%
Reddy et al. [82]	Healthy vs. Diabetic	PPG [5 min] 100 [50/50] In-house	SVM	HRV	Accuracy: 82% Sensitivity: 84% Specificity: 80%
Nirala et al. [83]	Healthy vs. Diabetic	PPG [pulse] 135 In-house	SVM	Signal and derivatives parameters + eigenvalues	Accuracy: 97.87% Sensitivity: 98.78% Specificity: 96.6%
Hettiarachchi et al. [84]	Healthy vs. Diabetic	PPG [2, 1 s] 150 [83/52] Public [85]	LDA	Signal features + physio data	Accuracy: 83%
Qawqzeh et al. [86]	Healthy vs. Diabetic	PPG [-] 587 [-] In-house	Logistic Regression	Signal features + physio data	Accuracy: 92.3% Sensitivity: 70% Specificity: 96%
Prabha et al. [87]	Healthy vs. Pre diabetic vs. Diabetic	PPG [5 s] 217 [-] Public [88]	Xboost	MFCC + physio data	Accuracy: 99.93% Sensitivity: 99.93% Specificity: 99.94%
Prabha et al. [87]	Healthy vs. Pre diabetic vs. Diabetic	PPG [5 s] 217 [-] Public [88]	SVM	MFCC + physio data	Accuracy: 92.28% Sensitivity: 86% Specificity: 94%
Chu et al. [74]	5 levels diabetes risk	PPG, ECG [1 min] 2538 [1310/1228] In-house	Logistic regression	HRV + physio data	Accuracy: 90% Sensitivity: 92.5% Specificity: 88%

Machine learning methods for diabetes detection. ^a^: type of signal [signal length], total number of subjects [subjects for each class], type of database. SVM: support vector machine. ARMA: autoregressive moving average. SVD: single value decomposition. RF: random forest. LS-SVM: least-squares support vector machine. LDA: latent dirichlet allocation. DWT: discrete wavelet transform. R-R: R to R ECG wave time interval. IMF: intrinsic mode functions. MFCC: Mel frequency cepstral coefficients. HOS: higher order spectral.

**Table 6:** Machine learning methods for blood glucose estimation and detection.

Reference	Objective	Data Type ^a^	Approach	Feature	Main Outcome
Nuryani et al. [93]	Hypoglycemia detection (T1DM)	ECG [8 h] 5 In-house	Fuzzy SVM	HR, QT, TT	Sensitivity: 74.2% Specificity: 59%
Monte-Moreno [89]	Glucose level estimation (DM)	PPG [5 s] 410 [331/79] In-house	RF	Signal features + pyhisio data	r = 0.9 Clarke Error Grid [87.7% A; 10.3% B]
Ling et al. [94]	Hypoglycemia detection (T1DM)	ECG [10 h] 16 In-house	GA-FI to	HR, QT and their variations	Sensitivity: 75% Specificity: 50%
Lipponen et al. [95]	Hypoglycemia detection (T1DM)	ECG [5 min] 22 In-house	PCA	QT, RT amplitude ratio	15/22 correct detection
Nuryani et al. [96]	Hypoglycemia detection (T1DM)	ECG [8 h] 5 In-house	Swarm-based SVM	Signal features	Sensitivity: 70.9% Specificity: 81.5%
Ling et al. [92]	Hypoglycemia detection (T1DM)	ECG [10 h] 16 In-house	HPSOWM-based FRM	HR, QT	Sensitivity: 85.7% Specificity: 79.8%
Ling et al. [97]	Hypoglycemia detection (T1DM)	ECG [6 h] 16 In-house	Extreme learning algorith	Signal features	Sensitivity: 78% Specificity: 60%
Zhang et al. [98]	Glucose level estimation	PPG [-] 18 In-house	SVR with GA	Signal features + physio data	r = 0.97 RMSE = 1.58 Clarke Error Grid [100% A]
Usman et al. [99]	Glucose level estimation	PPG [-] 71 In-house	Logisitc Regression	Second derivative feature	Accuracy: 69% Sensitivity: 73% Specificity: 64.7%
Zhang et al. [100]	Glucose level estimation	PPG [10 s] 18 In-house	SVR with GA	Signal features + physio data	Clarke Error Grid [100% A]
Chowdhury et al. [101]	Glucose level estimation	PPG [60 s] 18 In-house	PCR	Signal and derivative features	SEP = 18.30 mg/dL Clarke Error Grid [82.6% A; 17.4% B]
Zhang et al. [90]	Glucose level estimation (3 levels)	PPG [pulse] 80 [50/30] In-house	GSVM	GMM features	Accuracy: 81.49% Sensitivity: 79.6% Specificity: 83.2%
Gupta et al. [102]	Glucose level estimation	PPG [-] - In-house	RF	Signal features + physio data	r = 0.81
Hina et al. [103]	Glucose level estimation	PPG [10 s] 200 In-house	Fine Gaussian SVR	Signal features	RMSE = 11.28 Clarke Error Grid [95% A]
Gupta et al. [104]	Glucose level estimation	PPG [3 s] 26 In-house	XGBoost	Signal features + physio data	r = 0.94 MAE = 8.31 mg/dL
Islam et al. [105]	Glucose level estimation	PPG [50 s] 52 In-house	PLS	Signal and derivative parameters	SEP = 17.02 mg/dL
Shamim et al. [106]	Glucose level estimation	PPG, ECG [2 min] 1 In-house	CART	HRV	ECG HRV scored the best result. RMSE = 30 mg/dL
Guzman et al. [107]	Glucose level estimation	PPG [10 min] 5 In-house	SVR	HRV, BMI, fatigue, DBP	MAE = 16.24 mg/dL
Susuana et al. [108]	Glucose level estimation	PPG [11 s] 4 In-house	EBTA	Raw signal	Accuracy: 98%
Cichosz et al. [91]	Hypoglicemia detection (T1DM)	ECG, CGM [5 min] 10 In-house	Mathematical prediction model	HRV, CGM data	Sensitivity: 79% Specificity: 99% AUC: 0.99 Lead time: 22 min improvement
Elvebakk et al. [109]	Hypoglicemia detection (T1DM)	ECG, Activity, NIR, bioimpedance, skin temperature [30 min] 15 In-house	Multi parameter model	Probability of changes	Accuracy: 88% Sensitivity: 95% Specificity: 96% AUC: 0.97

Machine learning methods for blood glucose estimation and detection. ^a^: type of signal [signal length], total number of subjects [subjects for each class], type of database. HPSOWM-based FRM: hybrid particle-swarmoptimization with wavelet-mutation-based fuzzy reasoning model. EBTA: ensemble bagged trees algorithm. PCA: principal component analysis. RF: random forest. SVR: support vector regression. SVM: support vector machine. GSVM: Gaussian support vector machine. GA: genetic algorithm. GSVM: Gaussian support vector machine. GA FIS: genetic algorithm with fuzzy inference system. CART: classification and regression trees. PLS: partial least squares regression. BMI: body mass index. HR: heart rate. AUC: area under the curve QT: Q to T ECG wave time interval. TT: T to T ECG wave time interval. GMM: Gaussian mixture model. DBP: diastolic blood pressure. CGM: continuous glucose monitoring.

**Table 7:** Machine learning methods for diabetes complications.

Reference	Objective	Data Type ^a^	Approach	Feature	Main Outcome
Jelinek et al. [110]	Healthy vs. Diabetic with CAN	ECG [20 min] 20 [-/20] In-house	GBMLS	HRV (MAF)	Sensitivity: 89% Specificity: 98%

Machine learning methods for diabetes complications. ^a^: type of signal [signal length], total number of subjects [subjects for each class], type of database. GBMLS: graph based machine learning system. MAF: multi scale Allan Factor.

**Table 8:** Deep learning methods for diabetes detection.

Reference	Objective	Data Type ^a^	Approach	Feature	Main Outcome
Swapna [115]	Healthy vs. Diabetic	ECG [10 min] 20 In-house	CNN + LSTM + SVM	Raw signal	Accuracy: 95.7%
Yildirim et al. [116]	Healthy vs. Diabetic	ECG [2 s] 30 [15/15] In-house	CNN	HR spectrogram	Accuracy: 97.62% Sensitivity: 100% Specificity: 96.72%
Panwar et al. [117]	Healthy vs. Diabetic	PPG [2.1 s] 217 [-] Public [85]	CNN	Raw signal	Accuracy: 99.8% Sensitivity: 99.8% Specificity: 99.8%
Avram et al. [112]	Healthy vs. Diabetic	PPG [21 s] 53870 [3584/50306] In-house	CNN + Logistic Regression	Raw signal+ physio data	Sensitivity: 75% Specificity: 65.4% AUC: 0.77
Wang et al. [113]	Healthy vs. Diabetic	ECG [5 s] - In-house	CNN	Raw signal+ physio data	Accuracy: 77.8% Sensitivity: 80.8% Specificity: 77.5% AUC: 0.77
Srinivasan et al. [114]	Healthy vs. Diabetic	PPG [30 s] 584 [467/341] Public [118]	CNN	Scalogram + physio data	Accuracy: 76.34% Sensitivity: 76.6% Specificity: 76.1% AUC: 0.83

Deep learning methods for diabetes detection. ^a^: type of signal [signal length], total number of subjects [subjects for each class], type of database. CNN: convolutional neural network. AUC: area under the curve HR: heart rate. LSTM: long short-term memory. SVM: support vector machine.

**Table 9:** Deep learning methods for blood glucose estimation and detection.

Reference	Objective	Data Type ^a^	Approach	Feature	Main Outcome
Nguyen et al. [122]	Hypoglycemia detection (T1DM)	ECG, GSR [4 h] 21 In-house	MLP	HR, QT length, skin impedance	Sensitivity: 95.2% Specificity: 41.4%
Nguyen et al. [123]	Hypoglycemia detection (T1DM)	ECG, GSR [4 h] 16 In-house	Bayesian neural network	HR, QT length, skin impedance	Sensitivity: 89.2% %
San et al. [124]	Hypoglycemia detection (T1DM)	ECG, GSR [10 h] 15 In-house	BBNN	HR, QT length, skin impedance	Sensitivity: 76.7% Specificity: 50.9%
San et al. [125]	Hypoglycemia detection (T1DM)	ECG, GSR [10 h] 15 In-house	ANFIS	HR, QT	Sensitivity: 79% Specificity: 51.8%
San et al. [126]	Hypoglycemia detection (T1DM)	ECG, GSR [10 h] 15 In-house	Rough BBNN	HR, QT and their variations	Sensitivity: 83.9% Specificity: 51.9%
Nguyen et al. [127]	Hyperglycemia detection (T1DM)	ECG [9h] 10 In-house	LM algorithm.	16 ECG parameters	Sensitivity: 70.6% Specificity: 65.4%
San et al. [128]	Hypoglycemia detection (T1DM)	ECG [10 h] 15 In-house	DBN	HR, QTc	Sensitivity: 79.7% Specificity: 50%
Manurung et al. [129]	Glucose level estimation	PPG [-] 51 In-house	MLP	Amplitude	MAE= 5.86 mg/dL
Hossain et al. [130]	Glucose level estimation	PPG [10 s] 30 In-house	CNN	Signal and derivative features features	r = 0.95 MSE = 0.15
Habbu et al. [119]	Glucose level estimation	PPG [1 min] 611 In-house	ANN	Time and frequency features	r = 0.84 Clarke Error Grid [80.6% A; 17.4% B]
Mahmud et al. [131]	Glucose level estimation	PPG, GSR, temperature [-] 15 In-house	CNN	Raw signal	Clarke Error Grid [80% A; 20% B]
Habbu et al. [119]	Glucose level estimation (T2DM)	PPG [1 min] 611 [378/233] In-house	MLP	CC	r = 0.95 Clarke Error Grid [85.2% A; 13.6% B]
Islam et al. [132]	Glucose level estimation (T2DM)	PPG, GSR [30 s] 25 [18/7] In-house	CNN	Raw signal	Clarke Error Grid [28% A; 43% B]
Porumb et al. [121]	Hypoglycemia detection	ECG, activity [5 min] 5 In-house	CNN + RNN	Raw signals	Accuracy: 82.4% Sensitivity: 86% Specificity: 80.6%
Cordeiro et al. [120]	Hypoglycemia detection	ECG [1 min] 1119 In-house	MLP	Signal features	Sensitivity: 87.6% Specificity: 85% AUC: 0.94

Deep learning methods blood glucose estimation and detection. ^a^: type of signal [signal length], total number of subjects [subjects for each class], type of database. MLP: multi layer perceptron. HR: heart rate. AUC: area under the curve QT: Q to T ECG wave time interval. DBN: deep belief network. BBN: block-based neural network. ANFIS: adaptive neural fuzzy inference system. CNN: convolutional neural network. LM: Levenberg–Marquardt. RNN: recurrent neural network. CC: cepstral coefficients.

**Table 10:** Deep learning methods for diabetes complications.

Reference	Objective	Data Type ^a^	Approach	Feature	Main Outcome
Alkhodari et al. [133]	Diabetic vs. Diabetic with complications	ECG [5 min] 95 [25/70] In-house	CNN	HRV	Accuracy: 98.5% Sensitivity: 100% Specificity: 97%

Deep learning methods for diabetes complications. ^a^: type of signal [signal length], total number of subjects [subjects for each class], type of database. CNN: convolutional neural network.

## Data Availability

The study did not report any data.
